# An experimental result of estimating an application volume by machine learning techniques

**DOI:** 10.1186/s40064-015-0791-3

**Published:** 2015-02-01

**Authors:** Tatsuhito Hasegawa, Makoto Koshino, Haruhiko Kimura

**Affiliations:** Graduate School of Natural Science and Technology, Kanazawa University, Kakumamachi, Kanazawa Japan; Department of Electronics and Information Engineering, Ishikawa National College of Technology, Kitachujo, Tsubatamachi Japan

**Keywords:** Smartphone, Machine learning, Context aware, Lifelog

## Abstract

In this study, we improved the usability of smartphones by automating a user’s operations. We developed an intelligent system using machine learning techniques that periodically detects a user’s context on a smartphone. We selected the Android operating system because it has the largest market share and highest flexibility of its development environment. In this paper, we describe an application that automatically adjusts application volume.

Adjusting the volume can be easily forgotten because users need to push the volume buttons to alter the volume depending on the given situation. Therefore, we developed an application that automatically adjusts the volume based on learned user settings. Application volume can be set differently from ringtone volume on Android devices, and these volume settings are associated with each specific application including games. Our application records a user’s location, the volume setting, the foreground application name and other such attributes as learning data, thereby estimating whether the volume should be adjusted using machine learning techniques via Weka.

**Electronic supplementary material:**

The online version of this article (doi:10.1186/s40064-015-0791-3) contains supplementary material, which is available to authorized users.

## Introduction

Use of mobile devices is remarkably growing all over the world. In 2013, according to a report from Strategy Analytics Inc.^a^, the smartphone market share continues to increase and has reached 60% globally. These smartphones have many functions, such as calling, e-mail, camera, games, media players, calendars, and alarm clocks. Thus, smartphones have almost the same abilities as personal computers and users always carry a smartphone. In addition, smartphones have many sensors as part of their standard equipment, unlike personal computers. Android smartphones generally have many sensors such as a light sensor, a microphone, an accelerometer, a gyroscope, and a magnetic field sensor. Android smartphones are suitable for recording lifelogs because they are equipped with many sensors, users always carry their smartphones, and smartphones offer high computing power similar to personal computers.

The term lifelog is composed of the words life and log; we define a lifelog as recorded individual behavior and information. As an example of a lifelog application, Hori and Aizawa (2003) and Sellen et al. (2007) have developed a system that records user’s memories, events, and experiences as a movie; therefore users can easily create such movies of their life stories. Up, a system developed by Jawbone^b^ can record our daily exercise and sleep patterns via an accelerometer installed in a wristband device; therefore users can use this recorded information to keep track of their health. Adami et al. (2003) used various types of sensors such that users could improve their quality of life via lifelog records. By simply recording the user’s location, we can develop an activity management system. Lifelogs are often manually recorded by user; however, it is generally desirable for the system to automatically record such information. To this end, although users should carry many devices on their, such as sensors and cameras, it makes users’ burdensome. In contrast, smartphone makes a much smaller burden for users because various sensors are readily available, and users always carry such devices of which lifelogs can automatically be recorded. Therefore, smartphones are suitable devices for recording lifelogs.

A smartphone is useful for users’ daily life; however, manners and morals of users have become a problem. For example, if someone played a video game at a loud volume in a public library, others may get disturbed. Setting the volume control of a smartphone is generally necessary in public places such as public libraries, theater, and so on. Moreover, in one’s social life, such as in school and at the workplace, it is desirable to set a suitable volume; however, a user needs to manually adjust the volume setting. Some users intentionally do not adjust volume settings depending on their morals, whereas others often forget to adjust the volume accordingly. This problem not only causes disturbances in the user’s surroundings, but also adversely affects a user’s own performance (Cutrell et al. 2001). If a user’s smartphone makes loud sounds during a class because he/she forgot to set it to silent mode, it not only causes confusion but also interrupts the class. This may also adversely affect a user’s reputation. Unnecessary noise during work adversely affects work performance and may increase stress and the likelihood of operational errors (Eyrolle and Cellier 2000). Although sound volume is an important setting, users need to manually adjust it according to their situation; therefore, automatic sound adjusting system is desirable.

In this study, we propose a system that automatically switches application volume ON or OFF for Android smartphones via machine learning techniques using users’ lifelogs. Furthermore, application volume can be set differently from ringtone volume on Android smartphone. There are two key reasons why we focused on application volume. First, application volume is not associated with silent mode preference, whereas ring tone volume is connected with silent mode preference. More specifically, unless application volume is set to zero, the smartphone makes noise even if the silent mode preference is set to silent, and this is uncomfortable for an inexperienced smartphone user. Second, there are users who adjust application volume on a regular basis without switching their silent mode preferences. In our experiment, five out of nine subjects adjusted application volume on a regular basis, but only one subject switched silent mode preference on a regular basis. Our nine subjects were composed of postgraduates, undergraduates and office workers. Because of the small number of subjects, we cannot be certain that most smartphone users do not switch silent mode preferences on a regular basis: however, users who do not set silent mode preferences do tend to adjust application volume on a regular basis. For this study, focusing on the volume settings of a smartphone, we propose an intelligent system that automatically sets suitable application volume by first learning a user’s ordinary volume setting patterns. Because an automatic application volume setting system prevents the smartphone from suddenly making noise, we achieve improved smartphone usability. Our proposed system runs in the background of an Android smartphone, constantly records terminal operational logs, and learns from these recorded logs. While changing foreground applications, a suitable application volume is estimated and automatically set. In general, application volume can be set to one of sixteen-gradations; however, proposed system estimates whether application volume is ON or OFF as the first step because the objective of this study is to prevent extreme negative influence for users and disturbance to their surroundings.

## Related work

We investigated studies related to our proposed system, including a study that enables users to automatically switch silent mode preferences and a study that prevents unwanted interruptions from incoming calls.

### Users’ lifelog

A lifelog is recorded individual behavior or information. We consider a user’s lifelog to be an individual’s behavior or information observed by a smartphone alone (i.e., without any other special devices). Various studies have focused on lifelogs observed by smartphones (Kobayashi et al. 2011; Kwapisz et al. 2010; Lee and Cho 2011). In these studies, systems inferred the user’s movement states by using analysis and classification of values observed via a three-dimensional accelerometer. In particular, in Kobayashi et al. (2011), successfully inferred how a user moves, including walking, running, riding a bicycle, stopping, driving a car, riding a bus, and riding a train. Further, we can also observe a user’s state via new activity recognition APIs on Android by Google. These studies make it possible to track how the user moves and are active as the user’s lifelogs. Other studies also make it possible to record various types of users’ lifelog information. Ouchi and Doi (2012) focused on indoor activity recognition, inferring an indoor activity such as cleaning or brushing one’s teeth. Chen et al. (2013) and Hao et al. (2013) measured sleep duration by using values observed on a smartphone’s three-dimensional accelerometer. We consider user’s lifelogs to include these user states inferred from sensor values as well as location information acquired via GPS, environmental noisiness, and the smartphone’s state (e.g., foreground application name, silent mode state, screen orientation, and Wi-Fi connection state); all such information as well as timing of such information is important to the composition of lifelogs.

### Sound volume setting

**Figure 1:**
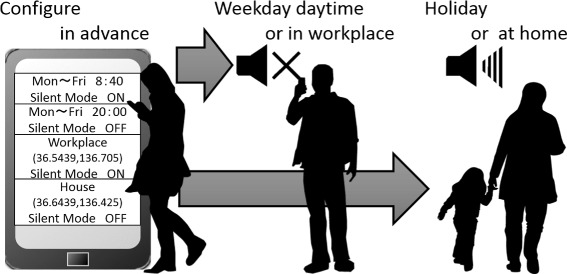
**Switching silent mode depending on user-defined terms set in advance.**

In 2014, automatic silent mode switching applications, such as AutoManner+^c^ and Silence^d^ were available on Google Play^e^ as Android applications. These applications are similar to an alarm clock, as illustrated in Figure 1; the applications switch silent mode preferences depending on terms set by the user in advance (e.g., day of the week, and time). HexRinger^f^, which is similar to these applications, can be set based on the day of the week, time, as well as location. This application switches silent mode preferences when the user enters an area configured by the user in advance. According to the number of application downloads, there indeed is a demand for applications that automatically change terminal preferences. Unfortunately, since users need to manually set these specific terms in such applications, the configuration is considered inconvenient. Moreover, it can be difficult for users who do not necessarily pay attention and understand their own regular behavior to configure these applications.

A study using an alternative approach was proposed by Khail and Connelly (2005), who proposed a method that automatically switches silent mode preferences depending on calendar information. This method learns silent mode preferences that depend on calendar information, including meetings, lunch dates, and shopping excursions, set by the user in advance. They analyzed the relationship between calendar information and silent mode preferences through an experiment in which users input suitable silent mode preferences using the PDA terminal as a cell phone while receiving incoming calls in daily life. The experimental results indicate that silent mode preferences could be set depending on calendar information to a certain degree; further, in the questionnaire, almost all subjects stated that they would like to use the system if it were available on their smartphone, although, there is no relationship between the users. Since this method focused on users who constantly switch silent mode preferences based on their schedule, this method may be suitable for users who can minutely update their calendars. In (Dekel et al. 2009), by using additional information, such as the day of the week, time, and the active profile, suitable silent profiles (e.g., general, silent, meeting, outdoor, and pager) are estimated using the k-nearest neighbor algorithm.

Context awareness is a research field related to our study. Context awareness is defined as a technology or concept in which a computer observes contexts such as users’ behavior, surrounding environment, and situations. From (Abowd et al. 1999) context awareness service is a general system that provides relevant services to users by automatically observing contexts without input from the user; because it provides a service by observing the user’s current situation and environment, it is often studied with mobile devices that are always or often carried by their users. Previously, a context-aware computer had to be connected to other sensing devices to collect contexts (Gellersen et al. 2002; Ho and Intille 2005), because sensing technologies on mobile devices had not yet been developed. Recent smartphones (e.g., Android and iPhone) are equipped with various sensors as standard functions, and have, therefore, been easily able to automatically collect contextual information without using other devices.

In this study, we prevent disturbance to those in the surroundings and prevent decrease in user performance due to users forgetting to adjust the application volume. Therefore, we developed an automatic application volume setting system by learning contexts collected by using context-awareness techniques. As described above, related work regarding automatically switching silent mode preferences has been investigated using various methods; in most of these methods, users need to set specific criteria terms such as time, location, and detailed schedules in advance. Conversely, in this study, we provide a feature for automatically setting application volume by learning contexts collected by a smartphone, thereby gradually learning suitable volume settings without requirng additional user inputs such as time, location and schedule in advance. Moreover, unlike much of the related work, we define a correct setting based on a user’s daily operations without having the user input correct (or incorrect) settings, thereby preventing trouble for the user’s volume settings, forgetting application volume settings, causing surrounding disturbances, and decreasing the user’s work performance.

## Automatic adjusting application volume

**Figure 2:**
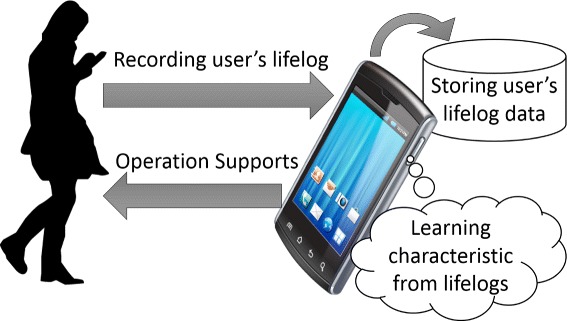
**Recording a user’s lifelog and applying it to support the user’s operations.**

In this study, we propose a method that automatically adjusts application volume via machine learning using contexts collected from one’s smartphone. The smartphone constantly records the user’s lifelogs, learns the user’s characteristics, and then estimates the suitable application volume for user’s operation supports, as illustrated in Figure 2. Moreover, our proposed application performs such as Additional file 1.

**Figure 3:**
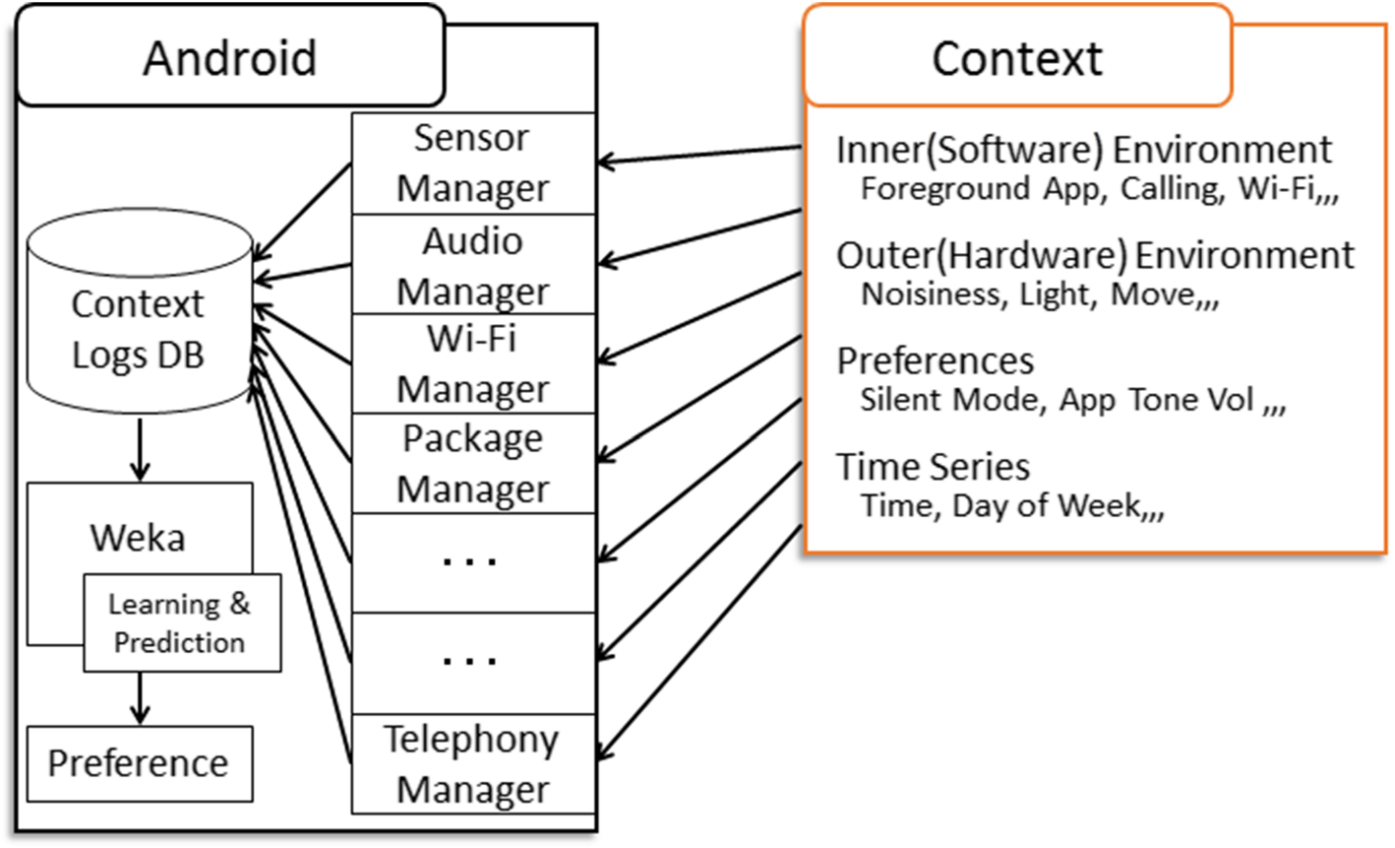
**Proposed system structure.**

More specifically, this system automatically sets application volume by extracting features from daily contexts such as the smartphone being used with application sound set to OFF in school and ON in the house. Our system automatically sets suitable volume settings by learning contexts recorded in the background. Figure 3 presents our system structure; from the figure, our system records various contexts through standard Android API managers such as SensorManager and AudioManager. Android smartphones can record various contexts that are roughly divided into the following types: Inner (Software) Environment, Outer (Hardware) Environment, Preferences, and Time Series. The inner environment is a smartphone’s software information and operational history including foreground application name, incoming call history, and Wi-Fi connected history. The outer environment is a user’s external environment including surrounding noise, location, brightness, and motion intensity. Preferences are the settings of a smartphone such as silent mode and application volume. Finally, the time series is time information such as the day of the week and time. These contexts are recorded in the context logs database. Using Weka, our system learns recorded contexts when the screen is turned off. Then, our system estimates whether application volume is ON or OFF when the foreground application is changed to automatically adjust the suitable application volume accordingly. Because of the excessive calculation load imposed when the screen is on that we decided to update classifier when the screen is turned off. Further, because it may confuse users if application volume suddenly adjusted during use, we decided to estimate whether application volume should be ON or OFF when changing the foreground application.

Our system records contexts in the background on a regular basis for learning. The information we decided to use as contexts is summarized in Table 1. Contexts denoted as for inner process are used for preprocessing described below and are not directly used as an attribute for machine learning. These contexts are recorded in the internal context logs database when an event shown in Table 2 occurs. Our system uses the Fused Location Provider setting by Google Play Services published in Google I/O (2013) for GPS measures. We set PRIORITY_BALANCED_POWER_ACCURACY as a priority setting and 1 minute as a measure interval time to limit battery consumption. Since low accuracy location was measured using this setting, unexpected and missing values were completed using preprocessing described below. GPS clustering is part of the preprocessing; more specifically, it is a result of clustering a latitude and longitude after complementation. We describe our clustering method below. Note that we selected these contexts because of hypothesis that they affect application volume settings.

**Table 1:** **Patterns of recorded data**

**Data pattern**	**Notes**
Screen on/off	Whether the screen is on or off
Screen orientation	Whether the displayed screen is landscape or portrait
Application name	Application name displayed in the foreground
Time	Time of day
Day of week	Day of week
Silent mode preference	What type of silent mode preference is set
Headset connection	Whether a headset is connected
GPS cluster	An index number of clustered GPS data
Datetime	(For inner process) A long value of datetime
Latitude	(For inner process) A latitude value observed with GPS
Longitude	(For inner process) A longitude value observed with GPS
GPS accuracy	(For inner process) An accuracy value observed with GPS
Application volume	(For inner process) An application volume setting

**Table 2:** **Timing of fetching data**

**Data fetching timing**	
Turning on the screen	Turning off the screen
Connecting the headset	Disconnecting the headset
Connecting the battery	Disconnecting the battery
Reacting to the proximity sensor	Losing reaction with the proximity sensor
Setting the silent mode	Canceling the silent mode
Dialing a phone call	Receiving an incoming call
Connecting a phone call	Returning to standby
Setting Wi-Fi on	Setting Wi-Fi off
Changing volume	Idle for 1 min
Charging a remaining battery	Pushing the home button

## Preprocessing

Before using recorded contexts as learning data for application volume estimation, they need to be preprocessed. One of the preprocessing steps involves GPS data, whereas another involves correct data generation for machine learning.

**Figure 4:**
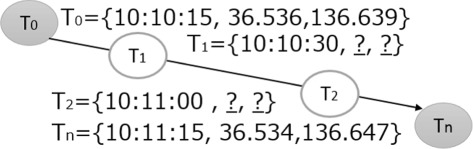
**Completion of unexpected or missing GPS data values.**

Above, we noted that our system uses the Fused Location Provider setting for GPS measures with PRIORITY_BALANCED_POWER_ACCURACY as a priority setting; however, using this setting, low accuracy data are often measured because this approach measures location by estimation using sensors, Wi-Fi, and a 3G connection with the minimum GPS measure to prevent increased battery consumption. Therefore, this low-accuracy GPS data must be completed. Since we considered it difficult to obtain suitable features from simple location information for application volume estimation, we decided to use a GPS cluster of significant places by clustering simple location information as an attribute. Unexpected and missing GPS value are completed using Euclidean distance with passed time and high accuracy data measured across target data under the assumption that such data progressed along g straight line. For instance, if *T*_0_ and *T*_*n*_ were measured and highly accurate and *T*_1_- *T*_*n*−1_ were unexpected or missing values, as shown in Figure 4, under the assumption that *T*_*n*_ has {Time, Latitude, Longitude} as an attribute, unexpected or missing values would be completed by the formula below. In the formula, Interval(a, b) is the time interval between a and b, whereas Distance(a, b) is the Euclidean distance between a and b. Note that these are completed if Interval(*T*_0_, *T*_*n*_) is within 5 min or within 1 h with Distance(*T*_0_, *T*_*n*_) within 50 m, then other cases are used as missing values. In general, we have:

$$\begin{array}{@{}rcl@{}} T_{i}=T_{0} + \frac{Interval(T_{i}, T_{0})}{Interval(T_{n}, T_{0})} \times Distance(T_{n}, T_{0}) \;.  \end{array} $$

Subsequently, our system preprocesses clustering data using completed latitude and longitude for extracting significant places. Significant places are meaningful areas that the user has visited, for example a user’s home, a station, and the workplace. There are numerous clustering algorithms to choose from, including k-means clustering, which is a non-hierarchical clustering method that divides a dataset into k clusters using a center-of-gravity approach; however, significant places cannot be identified by simply applying this algorithm to location information (i.e., latitude and longitude). In this study, we decided to use the time-based clustering method proposed by Kang et al. (2004), which labels locations as significant places when the distance from the measured location is less than a constant distance, and the measured interval is more than a constant interval time using a time series incidental to latitude and longitude. We decided to use this method because this algorithm is simple and processes data sequentially. Simplicity is especially important for development of mobile devices to ensure low battery consumption and little use of limited CPU resources. The time and distance threshold values are set to 300 s and 50 m respectively.

## Learning data

Next, our system generates a learning dataset, including correct data for machine learning. For estimation, correct application volume ideally should be constantly recorded by user; however, this imposes a significant load on the user. Therefore, our system uses past setting logs as correct data for machine learning. Because our system estimates application volume when changing the foreground application, a learning instance is generated for every application-change event. We defined the application-change event as the instant when the foreground application is changed and when the screen is turned on and off, exceptions being when 5 min have elapsed from the last-observed context. Table 3 shows a sample case in which instances for learning have been generated. Instances were generated at each line of the table (i.e., for lines 1-2, 3-10, 11-14, and 15-16). Correct application volume (i.e., ON or OFF) was decided by majority vote in the learning contexts. For example, in the second instance of Table 3 (i.e., lines 3-10), the initial application volume (i.e., lines 3-4) was ON; however, after line 5, it was set to OFF, and thereafter the correct data was recorded as OFF in the generated instance. Our system generates learning instances composed of attribute values and correct data from each learning context.

**Table 3:** **A portion of sample recorded data**

**Screen**	**Application**	**Time**	**GPS**	**Volume**	**Correct**
**On or Off**	**Name**		**Cluster**	**On or Off**	**Data**
ON	Home	1323	Place1	ON	True
ON	Home	1324	Place1	ON	True
ON	Puzzle	1325	Place1	ON	False
ON	Puzzle	1326	Place1	ON	False
ON	Puzzle	1327	Place1	OFF	False
ON	Puzzle	1328	Place1	OFF	False
ON	Puzzle	1329	Place1	OFF	False
ON	Puzzle	1330	Place1	OFF	False
ON	Puzzle	1331	Place1	OFF	False
ON	Puzzle	1332	Place1	OFF	False
ON	Home	1333	Place1	OFF	False
ON	Home	1334	Place1	OFF	False
ON	Home	1335	Missing	OFF	False
ON	Home	1336	Missing	OFF	False
OFF	Home	1337	Missing	OFF	False
OFF	Home	1338	Missing	OFF	False

## Experimental evaluation

Unlike the conventional application and related work, our system focuses on application volume estimation and does not require users to provide correct data inputs (e.g., time, location, and calendar) in advance. Therefore, it is difficult to simply compare our proposed system with related works having automatic silent mode setting. For this study, we showed our system’s usability by considering the accuracy with which our system can estimate the amount of burden our system can reduce. To evaluate accuracy of application volume estimation via our proposed system, we constructed an experiment to collect contexts from daily smartphone operations. For our experiment, we developed an application that records contexts (see Table 1) when specific events occur (see Table 2), and then sends recorded contexts to a web server on a regular basis. Since we can estimate users’ situations using observed contexts and what settings a user is using, we can analyze the amount of burden, caused by the users’ need to adjust application volume settings manually that this system can reduce. Since our application frequently records various contexts (e.g., GPS), battery consumption increased in this experiment, and it was difficult to convince users to participate in this experiment for a long time. For this experiment, we had nine participants, including undergraduates, postgraduates, office workers. The experimental period was 7-79 days, depending on the user, with an average period of 30 days per person. Among them, five users adjusted application volume on a regular basis; their experimental period was 13-79 days, with an average period of 41 days per person. Since only users who constantly adjust application volume constantly are the target audience for our proposed system, for evaluation accuracy, we analyzed data for only these five users. We measured estimation accuracy via the following steps: (1) we generated correct data using the steps described in Section 2; (2) we generated estimation results from our proposed system through a simulation that was the same as the actual application’s steps; and (3) we compared correct data with estimation results for evaluation. More specifically for (2), our simulation involved the following steps: (a) loading the recorded context one at a time; (b) preprocessing the complementation of GPS data and generating correct data; (c) estimating suitable application volume; (d) proceeding to the next context. Through simulation, we obtained results that were the same as actual data observed on the smartphone with actual usage.

## Classifier

Our system used machine learning techniques provided by the data mining software Weka for application volume estimation.

**Figure 5:**
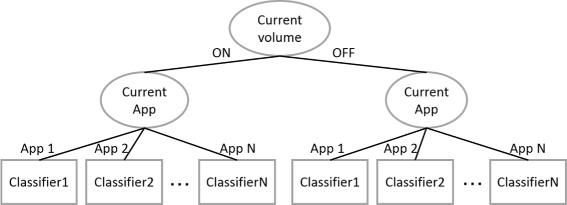
**Outline of a tree-structure classifier.**

The feature vectors for machine learning are eight attributes in Table 1 (i.e., excluding those denoted as for inner process"), and they are generated through the abovementioned preprocessing method. We hypothesized that the application volume setting notably depended on initial sound volume (i.e., ON or OFF) during estimaton and foreground application during estimation. Therefore, in this study, we constructed a tree-structure shown in Figure 5.

Further, to study suitable classifiers as leaf nodes in Figure 5, we compared the accuracies calculated by simulation using the following types of classifiers: random Forest (RF), support vector machine (SVM), naive Bayes (NB), PART. RF (Breiman 2001) is a method that calculates a result by majority vote in the generated trees’ classified results. SVM (Cortes and Vapnik 1995) is a supervised learning model that can efficiently perform nonlinear classification using a technique named kernel trick, implicitly mapping inputs into high-dimensional feature spaces. NB (Lewis 1998) is a simple probabilistic classifier based on Bayes’ theorem with strong independence assumptions. PART (Frank and Witten 1998) is a rule-learning classifier that infers rules by repeatedly generating partial decision trees, thus combining the two major paradigms for rule generation, creating rules from decision trees and the separate-and-conquer technique. We selected these algorithms because of their high accuracy and well-established learning models. We used these algorithms with following parameters which are initial values on Weka. In RF, maximum depth of the tree and the number of features were not limited, and the number of trees was 10. In SVM, the kernel function was RBF kernel, the cost was 1.0, and the gamma was 0.0. In NB, kernel estimator was unavailable. In PART, the minimum number of objects for making rules was two instances, pruning was unavailable.

## Evaluation criteria

We used the following criteria from Table 4 to evaluate our system Metsis et al. (2006). In this paper, we defined that True means that the application volume is on, and False means that the application volume is off.

$${\small\begin{aligned} Sensitivity & = \frac{TP}{TP+FN}\\ Specificity & = \frac{TN}{TN+FP}\\ PPV & = \frac{TP}{TP+FP}\\ NPV & = \frac{TN}{TN+FN}\\ Accuracy & = \frac{TP+TN}{TP+TN+FP+FN} \end{aligned}} $$

**Table 4:** **Evaluation indexes**

		**User adjusted**		
		**True**	**False**	
System adjusted	True	TP(True Positive)	FP(False Positive)	PPV
	False	FN(False Negative)	TN(True Negative)	NPV
		Sensitivity	Specificity	Accuracy

Sensitivity refers to the number of times that the system could automatically adjust the application volume on out of the number of times the user manually adjusted the application volume on (i.e., how much the system could estimate the application volume on). Specificity refers to the number of times that the system could automatically adjust the application volume off out of the number of times that the user manually adjusted the application volume off (i.e., how much the system could estimate the application volume off). Positive Predictive Value (PPV) refers to the number of times that the user wanted to adjust the application volume on out of the number of times that the system automatically adjusted application volume off (i.e., the rate of correct estimation of application volume on versus adjusted by the system). Negative Predictive Value (NPV) refers to the number of times that the user wanted to adjust the application volume off out of the number of times that the system automatically adjusted the application volume off (i.e., the rate of correct estimation the application volume off versus adjusted by the system). Accuracy refers to the rate of correct estimation of all. The higher the sensitivity, the fewer times the user needs to adjust the application volume on. The higher the specificity, the fewer times the user needs to adjust the application volume off.

To describe our proposed system’s usability, we compared our proposed system accuracy with the accuracy of the simple method where application volume is connected with silent mode preferences. We selected this method for comparison because it is easy to realize and can be used for simple application volume estimation.

## Experimental result

Table 5 shows the results of our evaluation. The simulation was performed on each user dataset. Table 5 indicates the average (Avg.) and standard deviation (SD) for each user in the form Avg. (±SD). Simple in the table indicates the accuracy of the method in which application volume is simply connected with silent mode preferences. RF, SVM, NB, and PART indicate the accuracy of our proposed system using each method as classifiers in Figure 5. Probability of occurrence the application volume on is 59.7%(±8.4%) in five subjects.

**Table 5:** **Comparison of accuracy between our proposed system and a simple method connected with silent mode preferences**

	**Sensitivity**	**Specificity**	**PPV**	**NPV**	**Accuracy**
Simple	52.0%(±41.9%)	99.9%(±0.1%)	99.4%(±0.7%)	78.0%(±18.6%)	80.1%(±17.2%)
RF	88.6%(±8.8%)	98.7%(±1.0%)	97.4%(±2.7%)	92.7%(±6.8%)	94.6%(±4.5%)
SVM	89.4%(±8.5%)	99.1%(±0.8%)	98.4%(±1.2%)	93.2%(±6.4%)	95.2%(±4.2%)
NB	88.6%(±9.1%)	98.6%(±1.0%)	97.3%(±2.7%)	92.8%(±6.6%)	94.6%(±4.4%)
PART	89.3%(±8.6%)	98.9%(±1.0%)	97.7%(±2.6%)	93.1%(±6.4%)	95.0%(±4.3%)

First, we compare Simple with our proposed system. In terms of accuracy, while Simple had 80.1% accuracy, our proposed system was 94.6%, 95.2%, 94.6% and 95.0%, consistently higher than Simple.

As a result, our proposed system could estimate more accurately than Simple. In terms of sensitivity and specificity, Simple had the highest specificity; however it had the lowest sensitivity. This indicates that Simple strongly related to the application volume off; however, only using simple method can not automatically adjusted when the user want to adjust the application volume on with silent mode on. In contrast, our proposed system can estimate both application volume on and off, moreover the specificity was not greatly different from Simple’s one. Therefore, our proposed system has better usability than Simple, because it hardly obstructs volume setting and often supports user volume setting. Second, we compared the accuracy of each classifier. There is no difference in each algorithm. We considered that the reason of this result is that we used the classifier with the structure shown in Figure 5.

Consequently, our proposed system can automatically switch application volume with higher accuracy than the method simply connected with silent mode preferences.

## Conclusions

The objective of this study was to support user operations by learning contexts observed on a smartphone. In this section, we described our proposed system that automatically adjusts application volume to prevent interruptions in the surrounding and decrease in user performance caused by forgetting to adjust application volume settings. Our proposed method estimates a suitable application volume and automatically adjusts it by observing and learning contexts on a regular basis. As a result of our experimental accuracy evaluation via simulation using actual contexts, we found that our proposed method could set application volume more effectively than the simple method connected with silent mode preferences. In terms of a classifier, we obtained the best accuracy through the tree structure based on initial sound volume and foreground application with the PART classifier.

To evaluate our proposed system through a simulation using contexts observed by actual usage, we considered the accuracy with which our system could estimate if the user utilized our proposed system. In this paper, we evaluated our method through a simulation. We will continue our work by improving estimation accuracy through developing an application that users all over the world can easily use; further, we will consider our proposed system usability and battery consumption by machine learning on the smartphone.

## Endnotes

^a^[Strategy Analytics Inc] http://www.strategyanalytics.com/

^b^[Up by Jawbone] https://jawbone.com/up

^c^[AutoManner+] https://play.google.com/store/apps/details?id=jp.chau2chaun2.mannerstimer

^d^[Silence] https://play.google.com/store/apps/details?id=net.epsilonlabs.silence.ads

^e^[Google Play] https://play.google.com/

^f^[HexRinger] https://play.google.com/store/apps/details?id=com.amay077.android.hexringer

## Additional file

Additional file 1
**An introduction movie of our proposed application.**

## References

[CR1] Abowd GD, Dey AK, Brown PJ, Davies N, Smith M, Steggles P (1999). Towards a better understanding of context and context-awareness. Comput Syst.

[CR2] Adami AM, Hayes TL, Pavel M (2003). Unobtrusive monitoring of sleep patterns. Proceedings of the 25th annual international conference of the IEEE engineering in medicine and biology society (IEEE Cat. No.03CH37439).

[CR3] Breiman L (2001). Random forests. Mach Learn.

[CR4] Chen Z, Lin M, Chen F, Lane ND, Cardone G, Wang R, Li T, Chen Y, Choudhury T, Campbell AT (2013). Unobtrusive sleep monitoring using smartphones. Pervasive computing technologies for healthcare (pervasive health) 2013 7th international conference on.

[CR5] Cortes C, Vapnik V (1995). Support-vector networks. Mach Learn.

[CR6] Cutrell E, Czerwinski M, Horvitz E (2001). Notification, disruption, and memory: Effects of messaging interruptions on memory and performance. Conference on human computer interaction (Interact 2001).

[CR7] Dekel A, Nacht D, Kirkpatrick S (2009). Minimizing mobile phone disruption via smart profile management. 11th international conference on human-computer interaction with mobile devices and services - MobileHCI ’09 vol 43.

[CR8] Eyrolle H, Cellier JM (2000). The effects of interruptions in work activity: Field and laboratory results. Appl Ergon.

[CR9] Frank E, Witten IH (1998). Generating accurate rule sets without global optimization. Machine Learning: proceedings of the fifteenth international conference.

[CR10] Gellersen HW, Schmidt A, Beigl M (2002). Multi-sensor context-awareness in mobile devices and smart artifacts. Mobile Netw Appl.

[CR11] Hao T, Xing G, Zhou G (2013). iSleep: Unobtrusive sleep quality monitoring using smartphones. Proceedings of the 11th ACM conference on embedded networked sensor systems.

[CR12] Hori T, Aizawa K (2003). Context-based video retrieval system for the life-log applications. Proceedings of the 5th ACM SIGMM international workshop on multimedia information retrieval - MIR ’03.

[CR13] Ho J, Intille SS (2005). Using context-aware computing to reduce the perceived burden of interruptions from mobile devices. Proceedings of the SIGCHI conference on human factors in computing systems CHI 05 Portland.

[CR14] Kang JH, Welbourne W, Stewart B, Borriello G (2004). Extracting places from traces of locations. 2nd ACM International Workshop on Wireless Mobile Applications and Services on WLAN Hotspots vol. 9.

[CR15] Khalil A, Connelly K (2005). Context-aware configuration: a study on improving cell phone awareness. Modeling and using context vol. 3554.

[CR16] Kobayashi A, Muramatsu S, Kamisaka D, Watanabe T, Minamikawa A, Iwamoto T, Yokoyama H (2011). Shaka: User movement estimation considering reliability, power saving, and latency using mobile phone. IEICE Trans Inf Syst.

[CR17] Kwapisz JR, Weiss GM, Moore SA (2010). Activity recognition using cell phone accelerometers. ACM SIGKDD Explorations Newsletter.

[CR18] Lewis DD (1998). Naive (Bayes) at forty: The independence assumption in information retrieval. Mach Learn: ECML-98.

[CR19] Lee YS, Cho SB (2011). Activity recognition using hierarchical hidden markov models on a smartphone with 3D accelerometer. Hybrid Artif Intell Syst.

[CR20] Metsis, V, Androutsopoulos I, Paliouras G (2006) Spam filtering with naive Bayes? which naive Bayes? In: Third conference on email and anti-spam (CEAS), California, USA.

[CR21] Ouchi K, Doi M (2012). Indoor-outdoor activity recognition by a smartphone. Proceedings of the 2012 ACM conference on ubiquitous computing - UbiComp ’12.

[CR22] Sellen A, Fogg A, Aitken M, Hodges S, Rother C, Wood K (2007). Do life-logging technologies support memory for the past? an experimental study using sensecam. Proceedings of the SIGCHI conference on human factors in computing systems.

